# Networking and Training in Palliative Care: Challenging Values and Changing Practice

**DOI:** 10.4103/0973-1075.76239

**Published:** 2011-01

**Authors:** Mhoira EF Leng

**Affiliations:** Makerere University, Kampala; Medical Director Cairdeas International Palliative Care Trust, United Kingdom

**Keywords:** Palliative care, Training, values, Professional practice

## Abstract

What make a good doctor is a question posed by the public and profession and is key when designing training programmes. The goal of training is to change practice not simply acquire knowledge yet too often curriculums and assessment focuses on knowledge and skills. Professional practice is underpinned by beliefs and values and therefore training may need to challenge deeply held values in order to result in a change in practice. Palliative care offers an opportunity to challenge values at a deeply personal level as it brings experiences of pain and suffering alongside clinical knowledge and skills. Palliative care is holistic and so real scenarios where physical, psychological, social and spiritual issues are evident can be presented in an interactive, learner centered environment. Training in ethics alongside clinical skills will assist the development of judgment which should also be assessed. Communication skills enable the clinician to hear and understand the needs and wishes of those facing life limiting illness. Training should include aspects of modeling and mentorship to demonstrate and integrate the learning with the realities of clinical practice and include those who lead and influence policy and advocacy.

## INTRODUCTION

’*I will be a better doctor now*’ commented a young trainee physician completing his palliative care attachment at Makerere University Palliative Care Unit. What prompted this statement? What does it mean to be a good doctor and how can palliative care training support new cadres of confident, ethical, skilled workers trained in the science and art of clinical care, as well as empower communities and families?

The concept of palliative care has emerged as part of a continuum that was historically the role of the family and community supported by traditions and faith. From the 20^th^ century, advances in medical care began to change the experience of end of life care with an increasingly medical and institutional focus. A recent survey in the UK reveals 66% of people would prefer to die at home yet 68% now die in hospital.[1] A needs assessment in an academic hospital in Uganda suggested that 45% of all patients on medical and surgical wards have a palliative care need.[2] Initiatives such as the Quality of Death index[3] seek to bring into sociological debate global compassions of care at the end of life and challenge reluctance to talk about death.

Most training curriculae for clinicians in India contain little or no palliative care training and this is reflected across the world.

*‘This palliative care course has reminded me why I came into medicine in the first place.’*

UK medical student

When we engage with colleagues in teaching and modeling palliative care we often witness a change in understanding which goes further than the acquisition of knowledge. Clinicians are usually highly motivated individuals who want to make a difference for their patients. They are taught and encouraged to cure and to save and even to prevent disease and suffering. Training focuses on the acquiring of a vast range of knowledge and competent skills which can be demonstrated and bring respect and affirmation. Yet when faced by the challenges of caring for people with life limiting illness, few options for cure, stretched resources and with the weight of expectations placed on young shoulders, health care workers care can become disillusioned, burnt out and feel a sense of disconnect between their values and experience.

**Figure 1:**
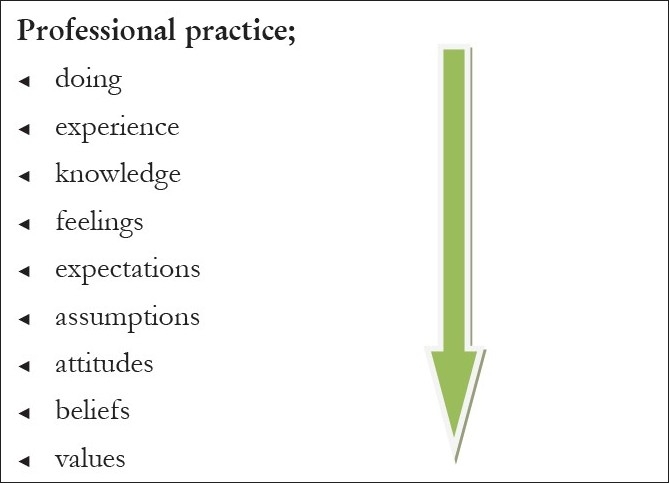
Underpinning of professional practice

Professional practice is rooted in values but overlaid with many other issues as represented in Figure 1.[4] Doing and experience are the surface aspects but will not change practice even if supported by new knowledge and skills if the deeper issues such as assumptions and values are not challenged. Training tends to focus on skills and knowledge and tries to include attitudes but less often addresses deeply held beliefs and values with the result we see little change in practice. Palliative care workers often tell of a significant personal experience from their family or professional life that challenged them and spurred them on to seek the knowledge and skills to make a difference. I remember clearly a man with lung cancer and ectopic ACTH secretion I met early in my career as a doctor. I kept asking what we could do to help his breathlessness and was told we would address this when we had corrected the electrolyte abnormalities and made a definitive diagnosis. Readers will not be surprised to know that the day we received these results my seniors congratulated me and asked how his breathing was now. I reported with sadness that he had died overnight and felt the helplessness and frustration of knowing we had done little to alleviate his symptoms or offer support. I lacked the understanding and the skills to adequately support him and his family. This experience was a personal prompt to seek training in palliative care more than twenty years ago and to have spent over a decade training others.

How can and does palliative care training challenge values? As we have seen educational theories underpinning professional practice remind us of the many layers separating doing and values. These need to be addressed and explored and palliative care with the mix of deeply human stories of pain and suffering, joys and courage alongside medical knowledge and therapeutic interventions is a rich source for self development and learning.

## FOCUS ON HOLISTIC CARE

‘*I feel better equipped to help people and their families.’ This will help my practice from tomorrow .*’

Students attending Palliative Care Toolkit training course

Holistic care underpins a palliative approach; encompassing the physical, social, psychological and spiritual dimensions. Dignity care,[5] a more recent paradigm brings another reminder of the essence of the human to human interaction that occurs when we come face to face with the prospect of life limiting illness. Training needs to address these dimensions and should be interactive and encourage learner directed discovery in a multi disciplinary context. The use of real scenarios or even better direct contact with those affected by life limiting illness ensures immediate relevance. Educational assessment should also reflect this approach including tools such as observed clinical practice and portfolio learning. The Palliative Care Toolkit[6] and associated Training Manual[7] is an excellent model for introducing palliative care principles utilizing this approach.

## JUDGEMENT

Many training programmes are now grappling with the challenge of teaching and assessing judgment and linking this with practice. The questions change from ‘What can we do?’ and “How can we do it?” but rather “What should we do?”and ‘Who should decide?’ Training in ethics combined with clinical skills may also help in recognising the concept of futility so pertinent in palliative care. Our best efforts and knowledge may not affect a cure but allow us to engage with patients and their families in addressing quality of life; in ‘putting life into their days not just days into their lives’.[8] This combination of practical reasoning harnessed to practical wisdom has been called the ‘artistry of practice’.[9]

## COMMUNICATION SKILLS

‘*I have learned to listen*.*Pastor* at spiritual care seminar

How do we know what will offer quality of life for a person and their family facing life limiting illness? How do we test out our judgments and practise the artistry? Palliative care is a partnership between health care workers and families and as with any relationship there needs to open, equal, meaningful and supportive communication. Areas such as active listening, breaking bad news, advanced care planning, and decisions about treatment options are essential topics, as are team dynamics and self awareness.

## MODELING AND MENTORSHIP

Many training programmes fail to demonstrate any change in practice due to a dissonance between what is taught and what the trainee sees in reality. Lack of support, little understanding about palliative care amongst seniors, low credibility and career opportunities for palliative care, over stretched capacity, failing health systems, lack of appropriate models of care and poor community engagement all contribute to difficulties in putting acquired learning into practice. In Uganda an audit of trained palliative care nurses revealed failures to implement their learning due to many of these factors.[10] Whilst I was leading a palliative care training course in India one of the participants, a young doctor, asked me to see a very ill young man who seemed to be in denial about his advanced oral cancer. There were complex social and medical issues and the team who were struggling to make good ethical decisions and feeling helpless in the face of unrealistic hope and even desperation. As we reviewed the situation and talked with the young man and his sister we acknowledged the challenges and feelings of helplessness, we explored ways forward and agreed the next steps. The young doctor thanked me and said ‘*I wanted to see if the theory you were teaching worked in practice so I took you to see our most difficult case’*. She needed to know if the training made sense out of the rarified atmosphere of the classroom and back into the real world of pain, suffering and limited options in her daily work.

## ADVOCACY AND POLICY

‘*I now understand what palliative care means and there will be a palliative care team in my hospital from this year’*.

Medical director of a rural hospital after a Palliative care Toolkit training course

We must ensure we engage the opinion leaders and policy makers at governmental, academic, institutional and community level. Many palliative care training programmes have successfully used public figures with wide popular appeal from the world of film, music, sport or even politics to raise awareness as well as funds. Holding a press conference, inviting key speakers to an inauguration or to present certificates can raise the profile and credibility of the training as well as raise awareness of palliative care. Also important to consider is the approval of any course by a credible academic authority and recognition by the relevant professional bodies. This in turn should be linked to service planning to ensure we train people with the right competencies for the right role who will be supported by policy, resources and planning to deliver the care that is needed.

Let me finish with a lesson from my own experience. When I came face to face with my own helplessness as a young doctor caring for the man who was so breathless and frightened it challenged me to address my own lack of skill. This man and his family however taught me a more important lesson. As I spoke with his family after the death expressing my sorrow and wondering if I should confess my feeling that we had failed to help, they thanked me saying ‘*Thank you for caring doctor; he felt safe when you visited.’* The value of presence above all.

## References

[1] Leadbeater C, Garber J (2011). Dying for a change. London: DEMOS think tank;.

[2] Lewington J (2011). Personal communication, abstract submitted EAPC. Europe: EAPC.

[3] The Quality of Death; Ranking end of life care across the world Economist Intelligence Unit.

[4] Coles C (1996). Approaching professional development. J Contin Educ Health Prof.

[5] Chochinov HM (2002). Dignity-conserving care-a new model for palliative care: Helping the patient feel valued. JAMA.

[6] Lavy V (2009). Palliative Care Toolkit Help the Hospices. ISBN;978-1-871978-71-1.

[7] Lavy V (2009). Palliative Care Toolkit Training Manual Help the Hospices.

[8] Wooldridge R (1991). Nairobi Hospice.Quoted in Lavy V. Palliative Care Toolkit p3.

[9] Fish D, Coles C (1997). Developing professional judgment in healthcare; learning through the critical appreciation of practice.

[10] Kiwanuka R (2009). An audit of palliative care services in Uganda. Uganda: Palliative Care Association of Uganda;.

